# Supplementary material for: The Secretome of Human Dental Pulp Stem Cells and Its Components GDF15 and HB-EGF Protect Amyotrophic Lateral Sclerosis Motoneurons against Death
Source: Biomedicines. 2023 Jul 30;11(8):2152. doi: 10.3390/biomedicines11082152 (PMC10452672; doi:10.3390/biomedicines11082152)
Supplement: Supplementary file 1 [file biomedicines-11-02152-s001.zip › Suplemental data/table1 supplemental data.docx]

**Table1 supplemental data : Statistical values**

**Fig1A :**

| **NTFs** | **-** | **-** | **-** | **-** | **-** | **-** | **-** | **+** |
| --- | --- | --- | --- | --- | --- | --- | --- | --- |
| **DPSCs-CM (%)** | **0** | **5** | **10** | **25** | **50** | **75** | **100** | **0** |
| **Mean±SEM (%)** | 100±0 | 108.6±17.4 | 137.6±18.4 | 244.6±38.4 | 218.1±14.3 | 251.0±38.9 | 172.6±26.9 | 265.9±18.6 |

**Fig1B :**

| **NTFs** | **-** | **-** | **-** | **+** |
| --- | --- | --- | --- | --- |
| **DPSCs-CM (%)** | **0** | **50** | **100** | **0** |
| **Mean±SEM (%)** | 100±0 | 123.1±10.19 | 100.9±18.95 | 332.1±65.85 |

**Fig1C :**

| **NTFs** | **-** | **-** | **-** | **+** |
| --- | --- | --- | --- | --- |
| **DPSCs-CM (%)** | **0** | **50** | **100** | **0** |
| **Mean±SEM (%)** | 100±0 | 119.7±22.96 | 137.3±24.85 | 289.1±58.17 |

**Fig1D :**

| **NTFs** | **-** | **-** | **+** |
| --- | --- | --- | --- |
| **DPSCs-CM (%)** | **0** | **50** | **0** |
| **Mean±SEM (%)** | 100±0 | 182.3±4.493 | 202.1±28.97 |

**Fig2B :**

| **NTFs** | **-** | **-** | **+** |
| --- | --- | --- | --- |
| **DPSCs-CM (%)** | **0** | **50** | **0** |
| **Mean±SEM (µm)** | 560.6±20.76 | 675.1±24.77 | 739.5±28.39 |

**Fig2C :**

| **NTFs** | **-** | **-** | **+** |
| --- | --- | --- | --- |
| **DPSCs-CM (%)** | **0** | **50** | **0** |
| **Mean±SEM (µm)** | 464.8±22.08 | 500.3±25.41 | 495.6±24.67 |

**Fig3B :**

| **NTFs** | **-** | **-** | **+** |
| --- | --- | --- | --- |
| **DPSCs-CM (%)** | **0** | **50** | **0** |
| **Mean±SEM(Hz)** | 2.438±0.3986 | 2.337±0.4365 | 2.018±0.3191 |

**Fig3C :**

| **NTFs** | **-** | **-** | **+** |
| --- | --- | --- | --- |
| **DPSCs-CM (%)** | **0** | **50** | **0** |
| **Mean±SEM(Hz)** | 2.443±0.3986 | 1.525±0.2607 | 2.248±0.3295 |

**Fig4A :**

| **NTFs** | **-** | **-** | **-** | **-** | **+** |
| --- | --- | --- | --- | --- | --- |
| **GDF15 (ng/ml)** | **0** | **10** | **50** | **100** | **0** |
| **Mean±SEM (%)** | 100±0 | 101.1±14.66 | 99.13±4.686 | 102.3±11.3 | 228.4±21.54 |

**Fig4B :**

| **NTFs** | **-** | **-** | **-** | **-** | **+** |
| --- | --- | --- | --- | --- | --- |
| **HB-EGF (ng/ml)** | **0** | **5** | **20** | **100** | **0** |
| **Mean±SEM (%)** | 100±0 | 98.49±8.49 | 88.09±6.56 | 92.54±5.69 | 198.8±11.79 |

**Fig4C :**

| **NTFs** | **-** | **-** | **-** | **+** |
| --- | --- | --- | --- | --- |
| **GDF15 (ng/ml)** | **0** | **10** | **0** | **0** |
| **HB-EGF (ng/ml)** | **0** | **0** | **20** | **0** |
| **Mean±SEM (%)** | 100±0 | 95.06±5.617 | 116.0±16.49 | 198.6±10.51 |

**Fig5A :**

| **DETANONOate 20 µM** | **-** | **-** | **-** | **+** | **+** | **+** |
| --- | --- | --- | --- | --- | --- | --- |
| **GDF15 (ng/ml)** | **-** | **10** | **-** | **-** | **10** | **-** |
| **HB-EGF (ng/ml)** | **-** | **-** | **20** | **-** | **-** | **20** |
| **Mean±SEM (%)** | 100±0 | 80.57±5.864 | 81.35±2.962 | 92.45±8.755 | 80.87±5.477 | 85.27±4.916 |

**Fig5B :**

| **DETANONOate 20 µM** | **-** | **-** | **-** | **+** | **+** | **+** |
| --- | --- | --- | --- | --- | --- | --- |
| **GDF15 (ng/ml)** | **-** | **10** | **-** | **-** | **10** | **-** |
| **HB-EGF (ng/ml)** | **-** | **-** | **20** | **-** | **-** | **20** |
| **Mean±SEM (%)** | 100±0 | 84.46±4.210 | 98.48±2.185 | 92.45±8.755 | 87.08±6.069 | 91.30±4.844 |

**Fig6A :**

| **mRNA expression levels normalized to wildtype spinal cord** | *Egfr* | *Erbb4* | *Tgfbr1* | *Tgfrbr2-v1* | *Tgfrbr2-v2* |
| --- | --- | --- | --- | --- | --- |
| **Mean±SEM (%)** | 1.00±0.00 | 0.60±0.05 | 1.13±0.61 | 1.39±0.58 | 1.74±0.82 |
|  |  |  |  |  |  |
