# Supplementary material for: The Secretome of Human Dental Pulp Stem Cells and Its Components GDF15 and HB-EGF Protect Amyotrophic Lateral Sclerosis Motoneurons against Death
Source: Biomedicines. 2023 Jul 30;11(8):2152. doi: 10.3390/biomedicines11082152 (PMC10452672; doi:10.3390/biomedicines11082152)
Supplement: Supplementary file 1 [file biomedicines-11-02152-s001.zip › Suplemental data/table2 supplemental data.docx]

**Table 2 supplemental data : statistical analysis**

| **expérimental conditions** | **statistical test (post hoc)** | ***P*** | ***F* (DFn. DFd)** |
| --- | --- | --- | --- |
| **Figure 1A** | one-way ANOVA (Tukey) | <0.0001 | *F*_(7.36)_ = 9.583 |
| negative control versus DPSCs-CM 5% |  | >0.9999 |  |
| negative control versus DPSCs-CM 10% |  | 0.9805 |  |
| negative control versus DPSCs-CM 25% |  | 0.001 |  |
| negative control versus DPSCs-CM 50% |  | 0.0008 |  |
| negative control versus DPSCs-CM 75% |  | 0.0005 |  |
| negative control versus DPSCs-CM 100% |  | 0.1624 |  |
| negative control versus positive control |  | <0.0001 |  |
| DPSCs-CM 5% versus DPSCs-CM 10% |  | 0.9991 |  |
| DPSCs-CM 5% versus DPSCs-CM 25% |  | 0.0734 |  |
| DPSCs-CM 5% versus DPSCs-CM 50% |  | 0.1581 |  |
| DPSCs-CM 5% versus DPSCs-CM 75% |  | 0.0524 |  |
| DPSCs-CM 5% versus DPSCs-CM 100% |  | 0.7898 |  |
| DPSCs-CM 5% versus positive control |  | 0.0084 |  |
| DPSCs-CM 10% versus DPSCs-CM 25% |  | 0.2714 |  |
| DPSCs-CM 10% versus DPSCs-CM 50% |  | 0.5083 |  |
| DPSCs-CM 10% versus DPSCs-CM 75% |  | 0.2093 |  |
| DPSCs-CM 10% versus DPSCs-CM 100% |  | 0.9898 |  |
| DPSCs-CM 10% versus positive control |  | 0.0531 |  |
| DPSCs-CM 25% versus DPSCs-CM 50% |  | 0.9894 |  |
| DPSCs-CM 25% versus DPSCs-CM 75% |  | >0.9999 |  |
| DPSCs-CM 25% versus DPSCs-CM 100% |  | 0.3953 |  |
| DPSCs-CM 25% versus positive control |  | 0.9968 |  |
| DPSCs-CM 50% versus DPSCs-CM 75% |  | 0.9639 |  |
| DPSCs-CM 50% versus DPSCs-CM 100% |  | 0.7256 |  |
| DPSCs-CM 50% versus positive control |  | 0.5528 |  |
| DPSCs-CM 75% versus DPSCs-CM 100% |  | 0.291 |  |
| DPSCs-CM 75% versus positive control |  | 0.9997 |  |
| DPSCs-CM 100% versus positive control |  | 0.0289 |  |
| **Figure 1B** | one-way ANOVA (Tukey) | 0.0037 | *F*_(3.8)_ = 10.56 |
| negative control versus DPSCs-CM 50% |  | 0.9631 |  |
| negative control versus DPSCs-CM 100% |  | >0.9999 |  |
| negative control versus positive control |  | 0.0064 |  |
| DPSCs-CM 50% versus DPSCs-CM 100% |  | 0.9671 |  |
| DPSCs-CM 50% versus positive control |  | 0.0117 |  |
| DPSCs-CM 100% versus positive control |  | 0.0065 |  |
| **Figure1C** | one-way ANOVA (Tukey) | 0.0095 | *F*_(3.12)_ = 6.036 |
| negative control versus DPSCs-CM 50% |  | 0.7049 |  |
| negative control versus DPSCs-CM 100% |  | 0.8605 |  |
| negative control versus positive control |  | 0.0086 |  |
| DPSCs-CM 50% versus DPSCs-CM 100% |  | 0.99 |  |
| DPSCs-CM 50% versus positive control |  | 0.0573 |  |
| DPSCs-CM 100% versus positive control |  | 0.034 |  |
|  |  |  |  |
| **expérimental conditions** | **statistical test (post hoc)** | ***P*** | ***F* (DFn. DFd)** |
| **Figure1D** | one-way ANOVA (Tukey) | 0.0048 | *F*_(2.9)_ = 10.24 |
| negative control versus DPSCs-CM 50% |  | 0.0183 |  |
| Negative control versus positive control |  | 0.0053 |  |
| DPSCs-CM 50% versus positive control |  | 0.6968 |  |
| **Figure2B** | one-way ANOVA (Tukey) | <0.0001 | *F*_(2. 399)_ = 13.18 |
| negative control versus DPSCs-CM 50% |  | 0.0036 |  |
| negative control versus positive control |  | <0.0001 |  |
| DPSCs-CM 50%versus positive control |  | 0.1586 |  |
| **Figure2C** | one-way ANOVA (Tukey) | 0.527 | *F*_(2. 402)_ = 0.6417 |
| negative control versus DPSCs-CM 50% |  | 0.6372 |  |
| negative control versus positive control |  | 0.5506 |  |
| DPSCs-CM 50%versus positive control |  | 0.9897 |  |
| **Figure3B** | one-way ANOVA (Tukey) | 0.689 | *F*_(2. 52)_ = 0.3752 |
| negative control versus DPSCs-CM 50% |  | 0.9832 |  |
| negative control versus positive control |  | 0.6991 |  |
| DPSCs-CM 50% versus positive control |  | 0.8196 |  |
| **Figure3C** | one-way ANOVA (Tukey) | 0.2157 | *F*_(2. 52)_ = 1.580 |
| negative control versus DPSCs-CM 50% |  | 0.2242 |  |
| negative control versus positive control |  | 0.9254 |  |
| DPSCs-CM 50% versus positive control |  | 0.3555 |  |
| **Figure4A** | one-way ANOVA (Tukey) | <0.0001 | *F*_(4. 10)_ = 19.73 |
| negative control versus GDF15 10ng/ml |  | >0.9999 |  |
| negative control versus GDF15 50ng/ml |  | >0.9999 |  |
| negative control versus GDF15 100ng/ml |  | >0.9999 |  |
| negative control versus positive control |  | 0.0003 |  |
| GDF15 10ng/ml versus GDF15 50ng/ml |  | >0.9999 |  |
| GDF15 10ng/ml versus GDF15 100ng/ml |  | >0.9999 |  |
| GDF15 10ng/ml versus positive control |  | 0.0003 |  |
| GDF15 50ng/ml versus GDF15 100ng/ml |  | 0.9998 |  |
| GDF15 50ng/ml versus positive control |  | 0.0002 |  |
| GDF15 100ng/ml versus positive control |  | 0.0003 |  |
| **Figure 4B** | one-way ANOVA (Tukey) | <0.0001 | *F*_(4. 15)_ = 38.12 |
| negative control versus HB-EGF 5ng/ml |  | 0.9999 |  |
| negative control versus HB-EGF 20ng/ml |  | 0.7978 |  |
| negative control versus HB-EGF 100ng/ml |  | 0.9542 |  |
| negative control versus positive control |  | <0.0001 |  |
| HB-EGF 5ng/ml versus HB-EGF 20ng/ml |  | 0.8641 |  |
| HB-EGF 5ng/ml versus HB-EGF 100ng/ml |  | 0.9796 |  |
| HB-EGF 5ng/ml versus positive control |  | <0.0001 |  |
| HB-EGF 20ng/ml versus HB-EGF 100ng/ml |  | 0.9931 |  |
| HB-EGF 20ng/ml versus positive control |  | <0.0001 |  |
| HB-EGF 100ng/ml versus positive control |  | <0.0001 |  |
|  |  |  |  |
| **expérimental conditions** | **statistical test (post hoc)** | ***P*** | ***F* (DFn. DFd)** |
| **Figure 4C** | one-way ANOVA (Tukey) | 0.0003 | *F*_(3. 8)_ = 22.54 |
| negative control versus GDF15 10ng/ml |  | 0.9851 |  |
| negative control versus HB-EGF 20ng/ml |  | 0.6918 |  |
| negative control versus positive control |  | 0.0006 |  |
| GDF15 10ng/ml versus HB-EGF 20ng/ml |  | 0.5025 |  |
| GDF15 10ng/ml versus positive control |  | 0.0004 |  |
| HB-EGF 20ng/ml versus positive control |  | 0.0019 |  |
| **Figure 5A** | one-way ANOVA (Tukey) | 0.1681 | *F*_(5. 10)_ = 1.976 |
| positive control versus GDF15 |  | 0.2843 |  |
| positive control versus. HBEGF |  | 0.3205 |  |
| positive control versus DETANONOate 20µM |  | 0.9107 |  |
| positive control versus DETANONOate 20µM + GDF15 10ng/ml |  | 0.208 |  |
| positive control versus DETANONOate 20µM + HB-EGF 20ng/ml |  | 0.437 |  |
| GDF15 10ng/ml versus HB-EGF 20 ng/ml |  | >0.9999 |  |
| GDF15 10ng/ml versus DETANONOate 20µM |  | 0.7301 |  |
| GDF15 10ng/ml versus DETANONOate 20µM + GDF15 10ng/ml |  | >0.9999 |  |
| GDF15 10ng/ml versus DETANONOate 20µM + HB-EGF 20ng/ml |  | 0.9922 |  |
| HB-EGF 20ng/ml versus DETANONOate 20µM |  | 0.7783 |  |
| HB-EGF 20ng/ml versus DETANONOate 20µM + GDF15 10ng/ml |  | >0.9999 |  |
| HB-EGF 20ng/ml versus DETANONOate 20µM + HB-EGF 20ng/ml |  | 0.9966 |  |
| DETANONOate 20µM versus DETANONOate 20µM + GDF15 10ng/ml |  | 0.6612 |  |
| DETANONOate 20µM versus DETANONOate 20µM + HB-EGF 20ng/ml |  | 0.9262 |  |
| DETANONOate 20µM + GDF15 10ng/ml versus  DETANONOate 20µM + HB-EGF 20ng/ml | | 0.9904 |  |
| **Figure 5B** | one-way ANOVA (Tukey) | 0.0005 | *F*_(5. 14)_ = 8.992 |
| positive control versus GDF15 |  | 0.2802 |  |
| positive control versus. HBEGF |  | >0.9999 |  |
| positive control versus DETANONOate 20µM |  | 0.0003 |  |
| positive control versus DETANONOate 20µM + GDF15 10ng/ml |  | 0.2643 |  |
| positive control versus DETANONOate 20µM + HB-EGF 20ng/ml |  | 0.6497 |  |
| GDF15 10ng/ml versus HB-EGF 20 ng/ml |  | 0.5242 |  |
| GDF15 10ng/ml versus DETANONOate 20µM |  | 0.1277 |  |
| GDF15 10ng/ml versus DETANONOate 20µM + GDF15 10ng/ml |  | 0.9988 |  |
| GDF15 10ng/ml versus DETANONOate 20µM + HB-EGF 20ng/ml |  | 0.9148 |  |
| HB-EGF 20ng/ml versus DETANONOate 20µM |  | 0.0033 |  |
| HB-EGF 20ng/ml versus DETANONOate 20µM + GDF15 10ng/ml |  | 0.5865 |  |
| HB-EGF 20ng/ml versus DETANONOate 20µM + HB-EGF 20ng/ml |  | 0.8985 |  |
| DETANONOate 20µM versus DETANONOate 20µM + GDF15 10ng/ml |  | 0.0186 |  |
| DETANONOate 20µM versus DETANONOate 20µM + HB-EGF 20ng/ml |  | 0.0047 |  |
| DETANONOate 20µM + GDF15 10ng/ml versus  DETANONOate 20µM + HB-EGF 20ng/ml | | 0.9724 |  |
|  |  |  |  |
| **expérimental conditions** | **statistical test (post hoc)** | ***p*** | ***U*** |
| **Figure 6A** | Mann Whitney test |  |  |
| *Egfr* SOD^G93A^ normalysed to  Wild type spinal cord |  | 0.46 | *U*_(25.00.11.00)_ = 5 |
| *Erbb4 SOD^G93A^ normalysed to*  *Wild type spinal cord* |  | 0.0079 | U_(40.00.15.00)_ = 0.0 |
| *Tgfbr1* SOD^G93A^ normalysed to  Wild type spinal cord |  | 0.0079 | U_(15.00.30.00)_ = 0.0 |
| *Tgfrbr2-v1* SOD^G93A^ normalysed to  Wild type spinal cord |  | >0.9999 | U_(25.00.20.00)_ = 10.00 |
| *Tgfrbr2-v2 SOD^G93A^ normalysed to*  *Wild type spinal cord* |  | >0.9999 | U_(22.50.13.50)_ = 7.500 |
|  |  |  |  |
